# Recovery and purification of acetic acid from extremely diluted solutions using a mixed bed ion exchange resin – technical feasibility

**DOI:** 10.1039/d4ra08341e

**Published:** 2025-01-03

**Authors:** Tomás Roncal, Ainhoa Aguirre, Yolanda Belaustegui, Elisabet Andrés

**Affiliations:** a TECNALIA, Basque Research and Technology Alliance (BRTA), Parque Tecnológico de San Sebastián Mikeletegi Pasealekua 2 20009 Donostia-San Sebastián Spain tomas.roncal@tecnalia.com +34 944 041 445 +34 946 430 850; b Parque Tecnológico de Álava Leonardo da Vinci 11 01510 Miñano Spain; c Parque Científico y Tecnológico de Bizkaia Astondo Bidea, Edif. 700 48160 Derio Spain

## Abstract

A downstream process for the recovery and purification of acetic acid (AA) from an extremely diluted solution (100 mg L^−1^) also containing a mixture of contaminating inorganic salts in the form of bicarbonates, phosphates, sulfates and chlorides (DPM medium) has been developed, showing its technical feasibility. The process involves two successive steps based on the use of a mixed bed ion exchange (IEX) resin. The first step, a demineralization treatment to remove the inorganic anions that could potentially interfere with the recovery and purification of AA, involves a combined treatment of calcium precipitation, acidification with the Amberlite IR-120 resin and treatment with the Amberlite MB20 mixed bed resin. This treatment allows the total removal of phosphate and sulfate (and likely bicarbonate) and 90% removal of chloride, while still retaining 91% of AA in solution. In the second step the demineralized medium is treated again with the Amberlite MB20 mixed bed resin in batch to completely remove AA and chloride remaining in solution and, finally, the anion-loaded resin is step-eluted with a low volume of diluted H_2_SO_4_ to selectively elute AA, obtaining a purified (68.5–82.2% recovery yield and 96.9–99.2% purity) and concentrated (>1500 mg L^−1^) solution of the acid.

## Abstract 2

A downstream process for the recovery, purification and concentration of acetic acid from an extremely diluted solution, involving demineralization, treatment with a mixed bed ion exchange resin and step-elution with H_2_SO_4_.

## Graphical abstract

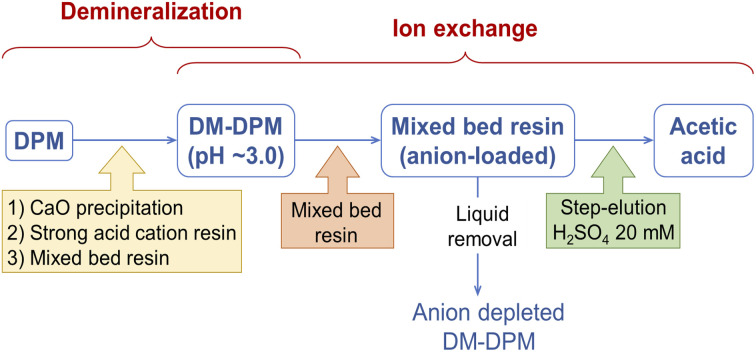

## 1. Introduction

The use of CO_2_ as a feedstock for producing chemicals through carbon capture and utilization (CCU) strategies is currently receiving a great deal of attention as a way to move towards a low carbon and circular economy.^1^ The great value of this approach is the possibility of valorising waste CO_2_ by converting it into industrially useful chemicals and polymers and, at the same time, reducing greenhouse gas emissions.^2^

One of the most interesting chemicals that can be obtained from CO_2_ is acetic acid (AA), a commodity chemical with many current uses in different industrial sectors (*i.e.*, textile, fibre, pharma, foods, *etc.*).^3,4^ The major consumption of AA comes from the synthesis of vinyl acetate monomer, used in the production of different polymers with applications as emulsifiers, resins, coatings, fibers and polymer wires. The other main use of AA is the production of cellulose acetate esters (through acetic anhydride). Moreover, glacial AA and some acetate esters are used extensively as solvents.

Industrial production of AA is dominated by thermocatalytic processes, including carbonylation of methanol and oxidation of acetaldehyde and hydrocarbons.^3^ Different chemo-catalytic methods for the conversion of CO_2_ into AA have been proposed, using either H_2_,^5^ methanol + H_2_,^6^ methane,^7^ or electricity^8^ to drive CO_2_ reduction. An interesting alternative to the chemical methods is based on biotechnology and involves the use of microorganisms or enzymes to catalyze that conversion. It is generally accepted that biotechnological methods show some advantageous properties when compared with chemical ones, such as ambient temperature and pressure operation, which reduces energy costs, and a high selectivity and specificity, which avoids byproduct generation. The main biological process for the conversion of CO_2_ into AA is carried out by autotrophic acetogenic bacteria and involves the Wood–Ljungdahl pathway.^9^ The main electron donors used by acetogens to drive reduction of CO_2_ are H_2_ and CO, but a wide range of organic compounds can also be used.^9^ Alternatively, electrons can also be supplied by electricity, through the so-called microbial electrosynthesis,^10^ or light, by means of organic semiconductor-bacteria biohybrid photosynthetic systems.^11^ Unfortunately, the industrial implementation of a biotechnological process for the conversion of CO_2_ into AA is currently a great challenge. The slow growth and low productivity of acetogens under autotrophic conditions, resulting from metabolic energy limitations, and the low solubility of gaseous substrates are important hurdles to overcome.^12^

A key factor to consider regarding AA and any other chemical's production processes, in general, is the need of recovering and purifying them through the so-called downstream processing. The aim of downstream processing is the efficient, reproducible, and safe recovery of the targeted product to the required specification (biological activity, purity, *etc.*), while maximizing recovery yield and minimizing costs. Product separation and purification from bioprocess media is often a complex task accounting for a significant share of the process costs (around 50–70%) of the total production cost can be attributed to it,^13^ mainly due to the low concentrations of the target molecules in the production media and the complexity of it. This is of special relevance for CO_2_ (gas)-derived products, that usually are present at much lower concentrations than, for example, their sugar-derived counterparts, so increasing downstream complexity and costs.^14^ Therefore, the development of efficient and cost-effective downstream processes for product recovery and purification is a mandatory need for industrial feasibility and accordingly, efficient, and non-energy intensive downstream technologies are preferred.

Recovery of AA from fermentation media has several key challenges, derived from its high solubility in aqueous media and its relatively low concentration. The concentration of AA in typical fermentation broths may vary over a wide range but is generally less than 10% by weight.^15^ Therefore, its recovery in pure form involves separation from a large quantity of water.

Different methods have been used for AA separation from fermentation broths, including distillation (simple, reactive, azeotropic, extractive), extraction or reactive extraction, supercritical fluid extraction, precipitation, crystallization, adsorption, ion-exchange, electrodialysis/electrodeionization, pressure-driven membrane methods, and pervaporation.^16,17^ Distillation and precipitation are the most conventional industrial methods, but they are neither economically nor environmentally feasible at the low concentrations found in fermentation broths. Furthermore, the presence of various ions (phosphate, chloride, sulfate, proteins) in significant amounts must be considered when designing a downstream separation process, since they could strongly interfere with the purification of AA.

Ion exchange (IEX) is found among the non-energy intensive technologies often used in downstream processing. IEX is a separation technique where insoluble polymers having different positively or negatively functional groups, called IEX resins, are used. These resins, normally present in the form of porous microbeads, membranes, or granules, have the potential to bind the ions of opposite charge. IEX, a separation process that do not require high power input, is widely used in bioseparations, in the recovery of organic acids, including AA, from aqueous fermentation media.^16–18^ Very often these IEX separation processes claim to address the recovery and purification of AA from diluted solutions but, when they talk about “diluted solutions”, they are referring to AA concentrations in the range 1–10 g L^−1^, that is, one to two orders of magnitude higher than the concentrations usually available in CO_2_-derived bioprocesses, which gives an idea of the extreme difficulty of the task.

In addition, purification of diluted carboxylic acids from bioprocess media using separation technologies based on electrical charge, such as IEX, poses a great challenge, not only due to the extremely low concentration of the target product, but also due to the complex composition of the media. Many of the different chemicals present in these media, mainly inorganic salts and low molecular weight charged organic compounds, often in concentrations higher than those of the carboxylic acid to be purified, can potentially interfere, by competition, with its purification, and would result in a lower recovery yield and a product having higher levels of impurities.

In the framework of the Horizon Europe Photo2Fuel project (https://www.photo2fuel.eu/), an artificial photosynthesis process for the conversion of CO_2_ into AA using a hybrid system of non-photosynthetic bacteria and organic photosensitisers is addressed, with sunlight as the only energy source.^11^ As the effluents obtained are characterized by the extremely low concentrations of AA, a suitable downstream processing should be developed not only to efficiently recover and purify the acid, but also to concentrate it. In this paper, such a downstream process is presented, involving the use of mixed bed IEX resins. The process is carried out in two steps: a first step to remove the contaminating mineral anions from the medium (demineralization), while AA remains in solution, and a second step to recover and concentrate AA from the mineral anions-free medium.

## 2. Materials and methods

### 2.1. Model solution

AA separation and purification experiments were carried out starting from DPM medium, a model solution with the following composition: 0.1 g L^−1^ AA, 0.4 g L^−1^ NaCl, 0.64 g L^−1^ K_2_HPO_4_, 1.5 g L^−1^ KH_2_PO_4_, 0.4 g L^−1^ NH_4_Cl, 0.33 g L^−1^ MgSO_4_·7H_2_O, 0.05 g L^−1^ CaCl_2_, 0.25 g L^−1^ KCl and 2.5 g L^−1^ NaHCO_3_. This model solution was based on defined photosynthesis medium (the actual DPM medium),^11^ the medium where artificial photosynthesis would be performed, but only containing its main inorganic salts. The amount of AA supplemented to this medium reflects the target concentration expected to be reached upon artificial photosynthesis. The minor components of the original DPM medium, trace mineral mix and Wolfe's vitamin mix, were omitted as they were considered not relevant for the different separation procedures to be applied. Although this model solution was slightly different from the original DPM medium this name was maintained in this work.

### 2.2. Ion exchange

IEX experiments were carried out either under batch mode or in column, using the IEX resins shown in Table 1. The resins were used as supplied, without any pretreatment. The quantities used refer to the mass as received (wet weight).

**Table 1:** IEX resins used in this work

IEX resin	Type	Functional group	Form
Amberlite MB20	Mixed bed strong acid cation and base anion	Sulfonic acid/trimethylammonium	H/OH
Amberlite IR-120	Strong acid cation	Sulfonic acid	H
Amberlite IRN78	Strong base anion	Trimethylammonium	OH
Amberlite IRA-67	Weak base anion	Tertiary amine	Free base
Lewatit VP OC 1065	Weak base anion	Primary amine	Free base

### 2.3. Ion chromatography

Concentration of AA (acetate) and inorganic anions (chloride, sulfate, and phosphate) was quantified by ion chromatography, using a Metrohm 930 Compact IC Flex ion chromatograph equipped with a conductivity detector. Anion separation was carried out in sequential suppressor mode on a Metrosep A Supp 19 – 250/4.0 analytical column connected in series with a Metrosep A Supp 19 Guard/4.0 precolumn. A gradient elution with the eluents A (4 mM Na_2_CO_3_) and B (20 mM Na_2_CO_3_) was used in the chromatographic separation as follows (flow rate, 0.7 mL min^−1^): eluent 100% A was initially held for 15 min, then this proportion was reduced to 20% in 25 min while that of B was increased from 0 to 80% and held for 10 min; finally, the proportion of B was reduced to zero while that of A increased to 100% in the next 0.1 min and held for 10 min. A solution of 500 mM H_2_SO_4_/100 mM oxalic acid/20% acetone was used as the regenerant. Column temperature was set at 35 °C and sample volume was 20 μL.

## 3. Results and discussion

### 3.1. Demineralization of DPM medium

#### 3.1.1. Demineralization of DPM medium by IEX with the resin Amberlite MB20

DPM medium, the medium from which AA is to be purified, without being a very complex medium, contains a mixture of salts in the form of bicarbonates, phosphates, sulfates, and chlorides. And, actually, AA is in minority with respect to the mineral anions: 100 mg L^−1^ AA, 1816 mg L^−1^ bicarbonate, 129 mg L^−1^ sulfate, 659 mg L^−1^ chloride and 1411 mg L^−1^ phosphate. As these inorganic anions could presumably interfere with the purification of AA through IEX^19^ a demineralization pretreatment of DPM medium was considered to be required to remove them and improve the subsequent purification of AA.

Demineralization of DPM medium was first addressed by IEX, using the Amberlite MB20 resin, a mixed bed resin containing both a strong acid cation exchange resin and a strong base anion exchange resin, supplied in the H and OH forms, respectively. This resin would allow the removal of both cations and anions in only one step. The chemical forms of the resin mean that cations in solution would be exchanged by protons (H^+^) in the resin and anions in solution would be exchanged by hydroxyl anions (OH^−^) in the resin. So, cation and anion binding to the resin would result in acidification and alkalinization of the solution, respectively. If the number of cation and anion equivalents bound to the resin are the same, so will the H^+^ and OH^−^ ions released, which would neutralize each other, and the pH of the solution should not be altered.

The key to demineralize the DPM medium with the IEX resin without, at the same time, also removing the AA, was to adjust medium to an acidic pH value well below its p*K*_a_, so that AA (a weak acid) is undissociated and, therefore, uncharged, while mineral anions remain still charged. This would consequently allow mineral anions to bind to the resin, but not AA, which would remain free in solution. Such kind of demineralization process was previously proposed for the purification of lactic acid from fermentation broths.^20^

According to its p*K*_a_ (4.76), 99.45% of AA would be undissociated at pH 2.5, so this pH was selected to perform the demineralization tests. Mineral anions would remain charged at this pH according to their p*K*a values: phosphoric acid p*K*_a1_ 2.12, sulfuric acid p*K*_a2_ 1.92, and hydrochloric acid p*K*_a_ −6.3. A very relevant difference would occur for carbonic acid (p*K*_a1_ 6.35) however, as will be explained at the end of this section.

**Fig. 1:**
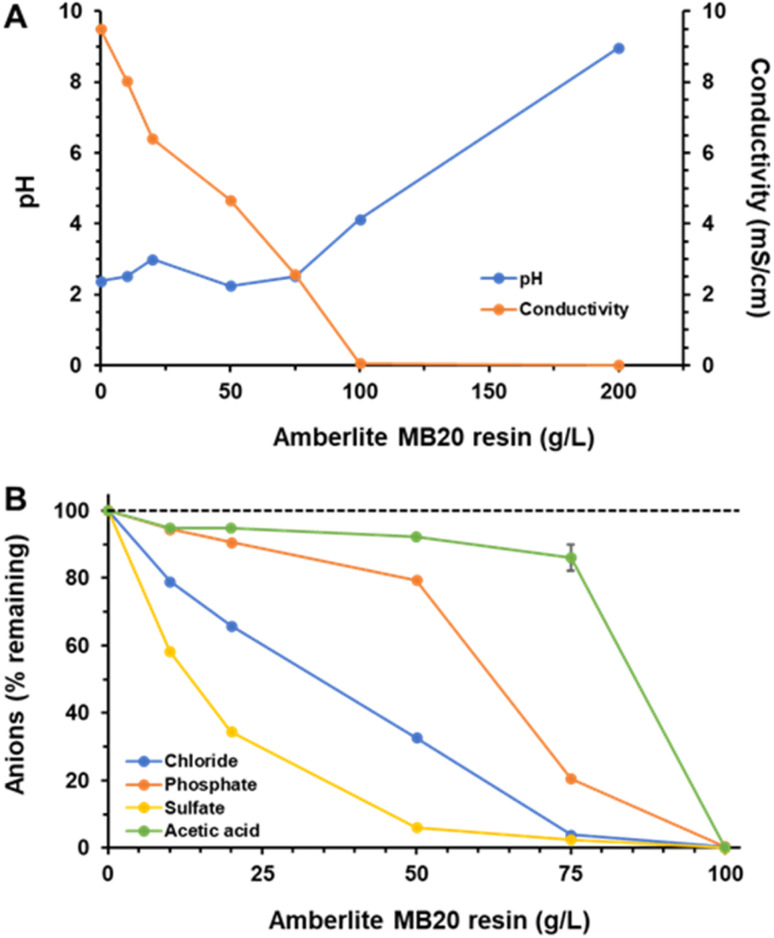
Treatment of DPM medium (pH 2.5) with different concentrations of the Amberlite MB20 resin (in g L^−1^ of medium). Equilibrium pH and conductivity (A) and amount of anions remaining in solution (B).

Consequently, HCl-acidified DPM medium (pH 2.5) was treated, in batch, with the Amberlite MB20 resin at different resin to medium ratios ranging from 10 to 200 g of resin per L of DPM medium. Results are shown in Fig. 1.

Two parameters, pH and conductivity at equilibrium, were directly measured to evaluate the effect of the treatments. pH remained practically unchanged around the initial pH of acidified DPM medium for ratios up to 75 g L^−1^ (Fig. 1A). At higher ratios medium pH increased, to around 4.1 at 100 g L^−1^ and near 9 at 200 g L^−1^.

As previously explained, if the same number of equivalents of cations and anions are bound to the resin, it was expected that the pH of the medium remained unchanged because the released H^+^ and OH^−^ ions would neutralize each other. Therefore, such pH increase would be the result of an unbalanced binding of cations and anions to Amberlite MB20, being higher that of the latter. According to the manufacturer, the percent volume of the anion exchange resin in Amberlite MB20 exceeds that of the cation exchange resin (62–56% *vs.* 38–44%). So, when the cation binding sites of the resin are saturated, more anions can still be bound, which would result in a net alkalinization of the solution.

Conductivity of DPM medium strongly decreased from its initial value of 9.5 mS cm^−1^ to virtually zero (60 μS cm^−1^) by increasing resin to medium ratio to 100 g L^−1^ (Fig. 1A). This decrease was quite linear and would reflect the removal of charged ions from the solution. So, apparently, the resin was very efficient in removing the salts.

When the salt composition of DPM medium after IEX resin treatment was analyzed (Fig. 1B) several conclusions could be extracted. First, the efficient removal of all the anions suggested by conductivity measurements was confirmed. All the anions, including acetate, were totally removed from DPM medium at 100 g L^−1^ resin. At a slightly lower resin to medium ratio, 75 g L^−1^, 97% of sulfate, 96% of chloride and 80% of phosphate were removed, while 86% of AA remained in solution. Second, the binding selectivity sequence of the anions to the resin, which reflects the affinity of the resin to them, was sulfate > chloride > phosphate (dihydrogen) > acetate, which agreed with the data reported in literature,^21^ so suggesting that this treatment could be very suitable to carry out demineralization of DPM medium because among the anions present the affinity of the resin for acetate (as free AA) was the lowest one.

However, as explained above, when the resin to medium ratio was higher than 100 g L^−1^, all the acetate was removed from the solution, which likely was a result of the parallel pH increase observed. The pH increase approaching and surpassing the p*K*_a_ of AA would displace equilibrium to the formation of the dissociated and charged acetate form, which could bind to the resin. So, it was very important that the pH of the medium during treatment with the IEX resin is maintained as far as possible in the acidic side from the p*K*_a_ of AA to avoid its removal.

Regarding the mineral anions, sulfate and chloride were almost totally removed at 75 g L^−1^ resin (97 and 96%, respectively). However, note that this value for the removal of chloride is related to an initial concentration of 2464 mg L^−1^, considerably higher than that contained in the original DPM medium (659 mg L^−1^), which is explained by the addition of HCl used to acidify DPM medium to the starting pH of 2.5. So, if the original chloride concentration is considered, the final 96 mg L^−1^ attained after resin treatment would represent a lower actual removal of 86%.

The most reluctant mineral anion to be removed was phosphate, remaining still 20% in solution at 75 g L^−1^ resin. By slightly increasing the ratio to 80 g L^−1^, the removal of phosphate increased to 88%, but then the AA remaining in solution decreased to 64% of the initial value, a loss that was considered unacceptable. The reason under the incomplete removal of phosphate is likely related to the different species found at equilibrium at the pH used (2.5). At this pH, the main species present in solution would be the monovalent dihydrogen phosphate anion (p*K*_a1_ 2.14), but around 30% of the undissociated and, therefore, uncharged form would be also present, and this later form could not bind to the resin.

Finally, some comments about bicarbonate, the most important mineral anion, in concentration terms, found in DPM medium. The ion chromatography method used to quantify the anions did not allow quantification of bicarbonate because the eluent used was Na_2_CO_3_. So, no direct information regarding this anion was available. However, we can suppose with a high degree of accuracy what happens with it. At the pH of the original DPM medium, around 7.1, bicarbonate is the main species found in solution (p*K*_a1_ 6.35), also appearing a fraction of undissociated carbonic acid. When pH is acidified to 2.5 before IEX resin treatment equilibrium would be totally shifted to the formation of carbonic acid, which, in turn, would decompose to CO_2_ and be released from solution as a gas. Therefore, it was expected that at the initial pH of the IEX resin treatment most of the bicarbonate anions would have been removed.

In conclusion, it appeared that the strategy used to demineralize DPM medium, without hardly affecting AA, using the mixed bed IEX resin Amberlite MB20 at a ratio of resin to medium of 75 g L^−1^ at acidic pH would be quite successful.

#### 3.1.2. Demineralization of DPM medium by calcium-precipitation and IEX with Amberlite MB20

The previous experiment, involving the treatment of DPM medium with the mixed bed IEX resin Amberlite MB20 at pH 2.5, allowed the almost complete removal of sulfate (and probably bicarbonate) anions, and most of chloride, still remaining around 86% of AA in solution. The problem was that more than 20% of phosphate still remained in the medium. So, a new strategy was planned to improve the demineralization extent, involving a calcium-treatment of DPM medium.

It is known that phosphate forms very insoluble salts with calcium. Therefore, treatment of phosphate-containing solutions with Ca^2+^ was expected to result in the precipitation of different calcium phosphate salts, so removing this anion from solution. The best option of calcium source was considered to be CaO (calcium oxide or quicklime), which is converted into Ca(OH)_2_ upon dissolution in water, because its use would have a double benefit. First, no extra anions would be added to the medium, avoiding the need to remove them later. And second, medium pH would become very alkaline, so favouring not only phosphate precipitation, but also bicarbonate removal, because at that pH values equilibrium would be displaced to the formation of carbonate anion (p*K*_a2_ 10.32), which would precipitate as the very insoluble CaCO_3_ salt.

Consequently, an amount of CaO sufficient to achieve 60 mM Ca^2+^ was added to the DPM medium and was let stirring overnight. This concentration of calcium is in excess from the bicarbonate and phosphate content of the medium, around 30 and 15 mM, respectively. From the solubility data of both calcium salts it was expected that calcium carbonate was precipitated first, and then calcium phosphate.

**Fig. 2:**
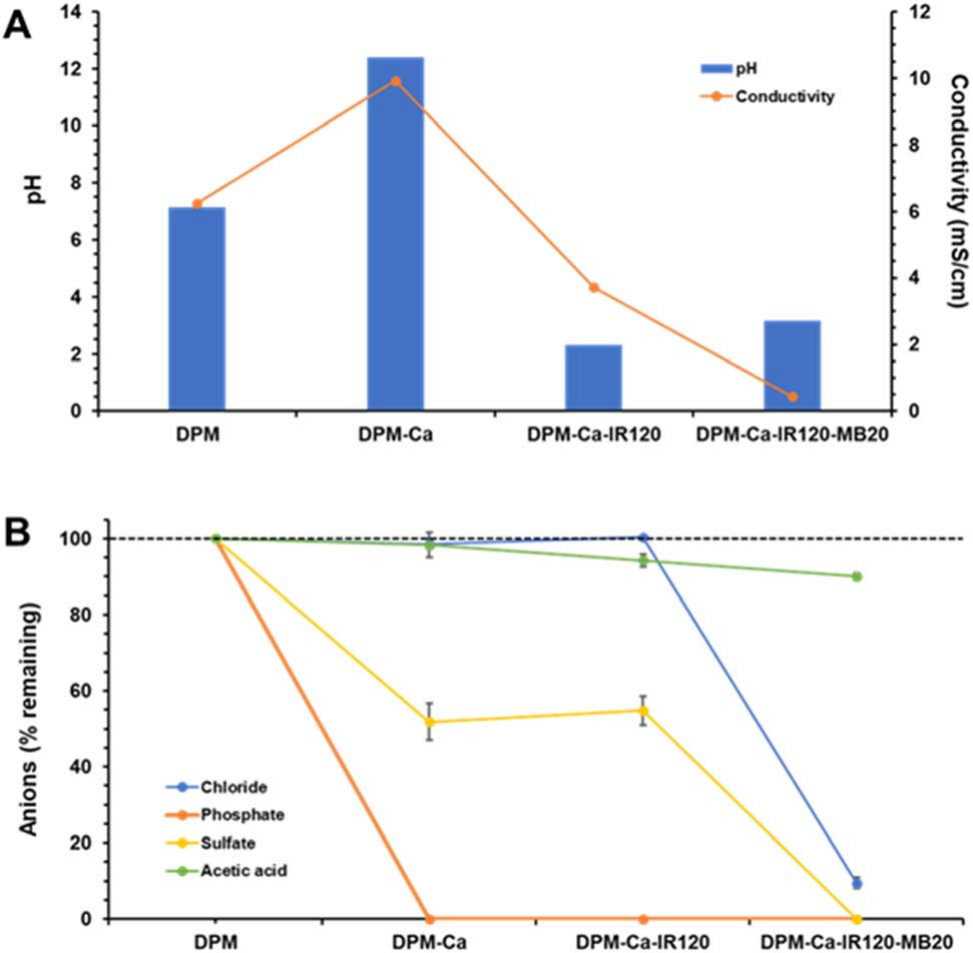
Combined treatment of DPM medium by calcium precipitation (DPM-Ca), acidification with the Amberlite IR-120 resin and treatment with the Amberlite MB20 resin. Equilibrium pH and conductivity (A) and amount of anions remaining in solution (B).

Following CaO addition a dense white precipitate appeared, rising the solution pH from an initial value of 7.14 to 12.40, and also increasing its conductivity from 6.24 to 9.92 mS cm^−1^ (Fig. 2A). The medium was then filtered to remove the precipitate, resulting in a clear filtrate with no trace of phosphate (Fig. 2B). Regarding bicarbonate, although it could not be quantified as explained before, it was also expected to be completely absent from the calcium-treated solution. Around half of sulfate, precipitated as gypsum, was also removed with the calcium treatment. The other anions, chloride and acetate, remained unchanged in solution.

So, once achieved the complete removal of phosphate, the most difficult mineral anion to be removed with the Amberlite MB20 resin at acidic pH, experiments of demineralization with this resin could be resumed. As explained before, the best pH to demineralize the medium with this resin, affecting AA as little as possible, was an acidic pH well below its p*K*_a_ value (4.76). So, the calcium-treated medium had to be acidified from its very alkaline pH of 12.40 to around 2.5 or less. One possibility to get such a strong acidification was to add strong mineral acids (*e.g.*, HCl or H_2_SO_4_), but this would result in an increase in the mineral anion content of the solution, which would complicate further treatment with the IEX resin. The most feasible alternative was the use of a strong acid cation exchange resin in the H form.^20^ Treatment of the calcium-precipitated DPM medium (DPM-Ca medium) with this type of resin would allow to remove the cations originally present in it and the excess of calcium likely still remaining after precipitation. And most importantly, the binding of these cations to the resin would be coupled to the release of an equivalent amount, in terms of charge, of protons, so resulting in a pH decrease of the solution.

According to these assumptions, DPM-Ca medium was treated with the strong acid cation exchange resin Amberlite IR-120 at a resin to medium ratio of 25 g L^−1^, a ratio sufficient to decrease the solution pH and conductivity to 2.30 and 3.72, respectively (Fig. 2A). This treatment, as expected, did not affect to the concentration of anions still present in solution, which remained unchanged (Fig. 2B), but strongly decreased the concentration of cations (results not shown). Regarding the anions, in the very unlikely event that there was still some trace of bicarbonate remaining in solution after calcium-precipitation, the strong acidification of the medium would have completed its removal, as it would have been released as CO_2_ gas.

Finally, this resin-acidified DPM-Ca medium was treated with the mixed bed IEX resin Amberlite MB20 to remove the remaining mineral anions (sulfate and chloride). When the resin to medium ratio was 20 g L^−1^ the pH increased from 2.30 to 3.15 and the conductivity decreased from 3.72 to 0.44 mS cm^−1^ (Fig. 2A). Under these conditions, while the AA concentration in solution was still kept at 91 mg L^−1^ (from the 100 mg L^−1^ in the original DPM medium), sulfate was completely removed, and chloride concentration decreased to 63 mg L^−1^ (from the initial 659 mg L^−1^) (Fig. 2B).

So, as a result of the combined treatments (calcium precipitation, acidification with the Amberlite IR-120 resin and treatment with the IEX resin Amberlite MB20) an almost complete demineralization of the DPM medium was achieved, with total removal of phosphate and sulfate (and likely bicarbonate) and 90% removal of chloride, while 91% of AA still remaining in solution.

A comparative summary of the results obtained with both demineralization treatments, with Amberlite MB20 alone and with the above combined treatment, is shown in Table 2. Results are shown in terms of concentration remaining in solution for four of the five main anions present in DPM medium. The values for the fifth anion, bicarbonate, are not shown because it could not be quantified by the ionic chromatography method used, but are expected to be zero or near zero according to the known behaviour of this anion under the different conditions applied to the medium (calcium-precipitation at alkaline pH and/or strong acidification). Although both treatments were very efficient in demineralizing DPM medium, but still maintaining most of the AA in solution, the combined treatment was somewhat better (Table 2).

**Table 2:** Concentration of anions remaining in solution, expressed as mg L^−1^ and % of the initial concentration, following demineralization treatments

Treatment	Chloride		Phosphate		Sulfate		Acetic acid	
	mg L^−1^	%	mg L^−1^	%	mg L^−1^	%	mg L^−1^	%
DPM (no treatment)^a^	659	100	1411	100	129	100	100	100
Amberlite MB20^b^	96	14.5	284	20.1	3	2.3	86	86
Combined treatment^c^	63	9.5	0	0	0	0	91	91

a Original DPM medium.

b Treatment with the IEX resin Amberlite MB20 alone at pH 2.5.

c Combined treatment: calcium-precipitation, acidification with Amberlite IR-120 and Amberlite MB20.

According to these results of the demineralization process of the DPM medium, a new synthetic simplified solution was prepared to be used in the next steps to purify AA. This synthetic simplified solution, called Demineralized-DPM (DM-DPM) medium, had the following composition: 91 mg L^−1^ AA and 63 mg L^−1^ chloride (as NaCl, 104 mg L^−1^), pH 3.15.

### 3.2. Recovery and purification of AA from DM-DPM medium by IEX

#### 3.2.1. Anion exchange resins comparison

**Fig. 3:**
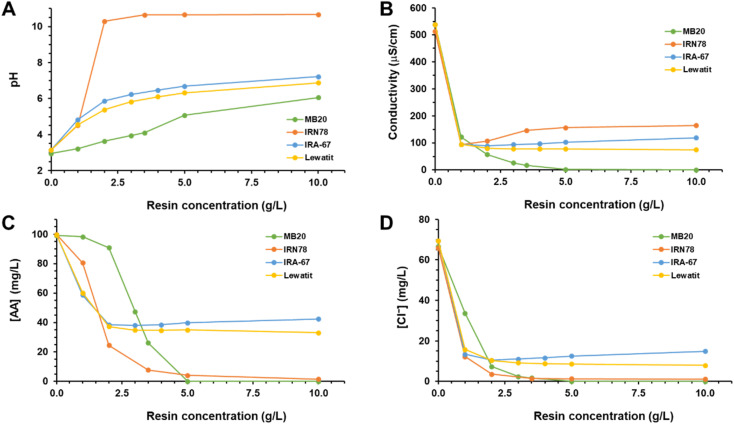
Treatment of DM-DPM medium with different anion exchange resins and the resulting medium pH (A), conductivity (B) and concentrations of AA (C) and chloride (D) at equilibrium.

Once DPM medium was almost completely demineralized, obtaining the DM-DPM medium as explained in the previous section, the recovery of AA from such an extremely diluted solution using IEX resins was addressed. For that, initially four different anion exchange resins were tested, covering all the types available in the market: two weak base anion (Amberlite IRA-67 and Lewatit VP OC 1065), one strong base anion (Amberlite IRN78) and one mixed bed strong acid cation and base anion (Amberlite MB20) resins. The resins were used as received, without any prior conditioning. DM-DPM medium was treated in batch with increasing concentrations of the single resins, ranging from 0 to 10 g L^−1^, and their capacity to remove AA and chloride from the solution was determined. In addition, changes in pH and conductivity were also recorded. The results of such assays are shown in Fig. 3.

First, weak base anion resins Amberlite IRA-67 and Lewatit VP OC 1065 had a very similar behaviour. Initially, by increasing the concentrations of the resins up to 2 g L^−1^, AA was increasingly removed from the starting 100 mg L^−1^, reaching its lowest concentration in the medium, around 35 mg L^−1^. From that point no additional AA was removed from the solution despite the increase in resin concentration to 10 g L^−1^. A similar effect was observed for chloride, that reached a minimum concentration of around 10 mg L^−1^ at 2 g L^−1^ resin. Regarding pH, it continuously increased in line with resin concentration from the initial value of 3.15 to around 7.00 at 10 g L^−1^ resin. Conductivity, in turn, strongly decreased to less than 100 μS cm^−1^ at the lowest concentration of resins assayed, stabilizing thereafter.

The reason behind the limited removal of AA by the weak base anion exchange resins can be probably found in the alkalinization of the medium resulting from the anion exchange activity of the resins, which increased pH to values higher than the p*K*_a_ of AA, so that its acid–base equilibrium was shifted to the formation of the charged acetate anion. And these weak base anion exchange resins, supplied in free base form, are known to only bind carboxylic acids as charge-neutral units (either through hydrogen bonding or *via* proton transfer) to maintain the charge neutrality of the adsorbent phase.^19,21^ In other words, they can only bind undissociated carboxylic acids, hence the importance of the pH being below the p*K*_a_ of the acid for a proper removal of it. Moreover, it should be noted that the mere presence of the resins in pure water caused a strong alkalinization to pH close to 9.0 (results not shown), which would be a consequence of the behavior as a weak base of their functional groups (free amines).

Amberlite IRN78 strong base anion exchange resin was more efficient for anions removal than the weak base resins, achieving values higher than 90 and 98% for AA and chloride, respectively, using resin concentrations higher than 3.5 g L^−1^. Medium pH rapidly rose to very alkaline values, higher than 10.0. As the functional group of the resin is in the OH form, the binding of anions results in the equivalent release of hydroxyl groups, the source of the alkalinization observed. However, unlike what happened with the weak base resins, in this case that alkalinization hardly affected to the extent of the anion binding. The functional group of this resin is trimethylammonium, so that it only binds dissociated, negatively charged, carboxylic acids, which are mainly found at alkaline pH values, when pH > p*K*_a_.

The comparison between weak and strong base anion exchange resins showed a higher AA removal capacity for the strong base resins, in agreement with other results found in the literature.^16^ However, the opposite trend has also been reported, *i.e.*, better performance of weak base resins compared to strong base ones.^22^ This discrepancy can probably be attributed to the different counter-ion present in the strong base anion exchange resins used in those studies. While in ref. 16 and the present work the resins were in the OH form, in ref. 22 they were in the Cl form. And it has been reported that the nature of the counter-ion in the resin influences strongly the exchange equilibrium, suggesting that the OH counter-ion is more easily displaced by the carboxylate anions than the Cl anion.^23^

Finally, Amberlite MB20, a mixed bed strong acid cation and base anion resin, behaved similarly to Amberlite IRN78, but with some relevant differences. The removal of AA and chloride was significantly lower with MB20 than with IRN78 at resin concentrations lower than 5 g L^−1^, but from this point on the removal was virtually complete with the former, while with the latter it was near but never reached. This difference could be explained by the fact that MB20 is a mixed bed resin, where only about half of it is a base anion resin. Therefore, at the same concentration of resin, the binding capacity of anions by MB20 would be lower (half, approximately) than by IRN78. This means that a two-fold concentration of MB20 would be needed, with respect to IRN78, to get the same result. However, although this explanation might be correct for chloride removal (Fig. 3D), it does not appear to be correct for AA (Fig. 3C).

The key for this discrepancy would be in the different pH evolution observed with both resins. As explained above, the use of the IRN78 resin resulted in a strong alkalinization of the medium upon anion binding. With the MB20 resin, however, the binding of the anions did not entail such strong pH increase, but it was better controlled (Fig. 3A). For resin concentrations lower than 5 g L^−1^, pH was maintained at values lower than the p*K*_a_ of AA, so that the acid was mainly in its undissociated uncharged form, which would not bind to the resin. Only from 5 g L^−1^ of resin the pH rose above the p*K*_a_ of the acid and, consequently, the concentration of the dissociated charged acetate anion, the species that actually binds to the resin, increased. The pH buffering capacity of the MB20 resin would result from the concerted activity of the base anion and acid cation resins present in it, so that the simultaneous binding of anions and cations would release hydroxyl groups and protons, respectively, that would neutralize each other. Therefore, the binding of AA, a weak acid, would depend on pH, which controls the proportion of acetate available to bind to the resin. On the contrary, as HCl is a strong acid, it is always completely dissociated and available to bind (chloride) independently of the pH.

It was previously mentioned that the removal of AA and chloride with the IRN78 resin was near to be complete but was never reached. This effect could result from the strong alkalinization induced upon anion binding, which means that the concentration of OH^−^ anions in the medium increased to such an extent that ultimately could compete for binding sites with the other anions. With the MB20 resin, as pH was better controlled, the concentration of OH^−^ anions would be very low and would not mean a real competition for the other anions, which could be removed completely.

In conclusion, from the above results it was considered that the best resin to address the recovery and purification of AA from the DM-DPM medium was the mixed bed resin Amberlite MB20 and, consequently, the next experiments were carried out using it.

#### 3.2.2. Recovery and purification of AA with Amberlite MB20 in batch

**Fig. 4:**
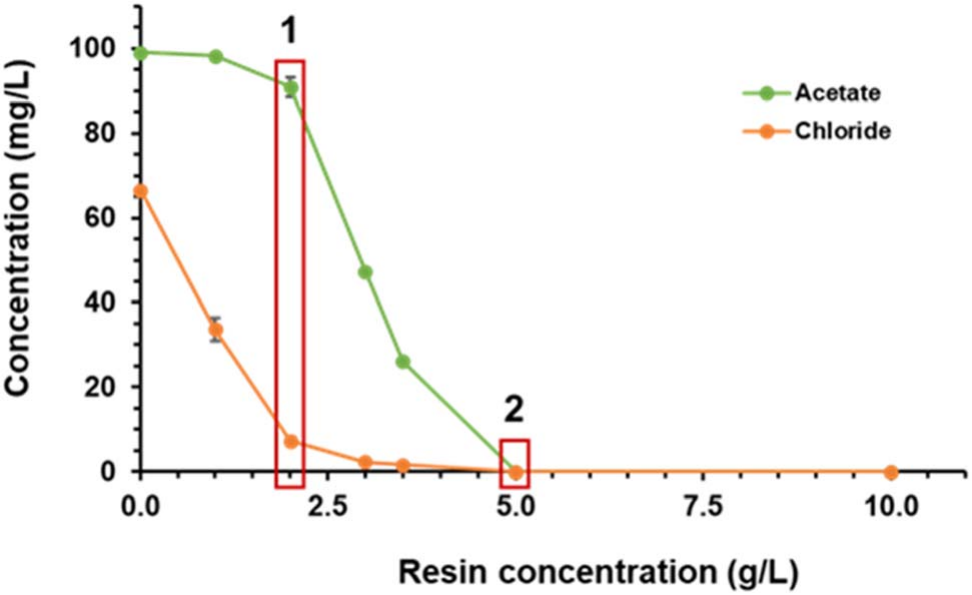
Removal of AA and chloride from DM-DPM medium by Amberlite MB20 at different resin concentrations.

Selecting from Fig. 3 the results related to the recovery of AA and chloride using the Amberlite MB20 resin and representing them in the same graph (Fig. 4) some interesting things can be observed.

An analysis of the graph in detail allowed to differentiate between two scenarios. In the first one (1 in Fig. 4), occurring at a resin concentration of 2 g L^−1^, the concentration of chloride was decreased from 67 mg L^−1^ (the concentration in DM-DPM medium) to 7.3 mg L^−1^, that is, it was decreased by 89% or, in other words, only 11% of the original chloride remained in solution. Meanwhile, AA concentration only decreased from 99 to 91 mg L^−1^, remaining in solution 92% of the initial acid. This means that at that resin concentration most of chloride was removed from DM-DPM medium, while most of AA remained in solution. So, in this scenario a “cleanup” of DM-DPM medium occurred, selectively removing chloride. As a result, the solution was relatively enriched in AA, so that its purity increased. In the second scenario (2 in Fig. 4), occurring at a resin concentration of 5 g L^−1^, both AA and chloride were totally removed from DM-DPM medium, so that it could be the starting point for alternative purification strategies where, following this first step, AA would be selectively released from the resin to separate it from the remaining chloride.

Several parameters can be used to evaluate the performance of the AA purification process:

(a) Yield (*Y*): mass percent of AA recovered in the process.

(b) Purity (*P*): mass percent of AA with respect to all the anions present in the medium.

(c) Purity increase (PI): ratio of the purity of AA obtained after any separation process with respect to its purity in the original DPM medium.

(d) Enrichment factor (EF): ratio of the concentrations (in mg L^−1^) of AA to the rest of anions in a sample with respect to its ratio in DPM medium.
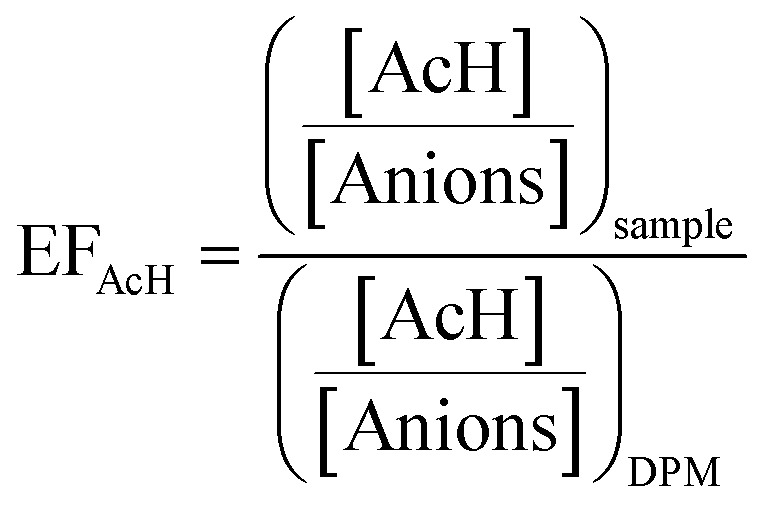


A summary of the performance of the AA purification process following scenario 1 of the treatment of DM-DPM medium with the Amberlite MB20 resin in batch is shown in Table 3. Therefore, after the treatment of DM-DPM medium with the Amberlite MB20 resin according to the conditions of scenario 1, a solution containing 83.7% of the AA present in the original DPM medium was obtained, with a purity of 92.6%, which represents a 38.6-fold purity increase and a 513-fold enrichment.

**Table 3:** Performance of the purification process of AA following scenario 1 of the treatment of DM-DPM medium with the Amberlite MB20 resin in batch or column

Parameter	DPM	DM-DPM	DM-DPM MB20 (batch)	DM-DPM MB20 (column)
Yield (%) step^a^	100	91.0	92.0	92.0
Yield (%) aggr.^b^	100	91.0	83.7	83.7
Purity (%)	2.4	59.8	92.6	80.4
Purity increase	1.0	24.6	38.6	33.5
Enrichment factor	1.0	58	513	164

a Yield in a step with respect to the previous one.

b Aggregate yield in a step with respect to the starting DPM medium.

#### 3.2.3. Recovery and purification of AA with Amberlite MB20 in column

In all the previous experiments, DM-DPM medium was treated with the Amberlite MB20 resin in batch. This operation mode involved the addition of a certain amount of resin to the medium, mixing for a sufficient contact time to achieve anions-resin binding equilibrium, and separation of resin and liquid fractions. There is an alternative operation mode where the medium is passed through the resin packed in a column, which might allow a better separation of AA and chloride. The resin binds chloride with higher affinity than AA, but under batch mode some AA is still removed, probably as a result of the pH increase observed. It would be possible that under column mode these pH changes could be better controlled, thus improving AA separation.

So, that column mode was tested aiming to selectively remove chloride from DM-DPM medium. As medium pH was acidic (2.95), a pH value where AA is undissociated and, therefore, uncharged, it was expected that it was unable to bind to the resin, while chloride, negatively charged, could do it. Therefore, the eluate could contain AA at the original concentration and be free of chloride. Once the resin had reached its maximum chloride binding capacity, it would begin eluting from the column.

**Fig. 5:**
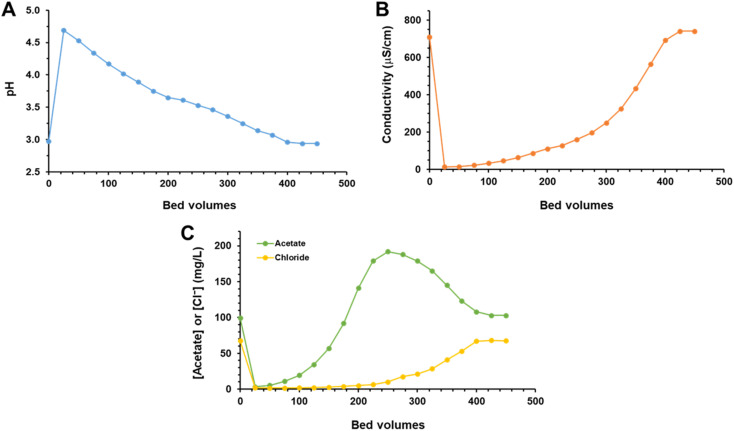
Breakthrough curve for AA and chloride contained in DM-DPM passed through a column of the Amberlite MB20 resin. pH (A), conductivity (B) and concentrations of AA and chloride (C) in the eluted fractions.

A column containing 0.65 g of Amberlite MB20, with a bed volume (BV) of 1 mL was prepared and the DM-DPM medium was passed through it with a flow rate of 1 mL min^−1^, equivalent to 60 BV per h. Fractions of 25 mL (25 BV) of the eluate were taken in the course of the experiment and characterized for the pH, conductivity and AA and chloride content. Results are shown in Fig. 5.

Initially, the eluate was almost free of AA. After a few BV, its concentration in the eluate started to increase, reaching a maximum value close to 200 mg L^−1^, double than in the feeding DM-DPM medium, at around 250 BV. Then, the AA concentration decreased to reach finally at about 400 BV the concentration present in the feeding solution and being unchanged thereafter. Most of chloride was, in turn, removed by the resin in the first 250 BV, maintaining a concentration in the eluate below 10 mg L^−1^, and then increased slowly to reach its feeding concentration by 400 BV. From that number of BV, the concentration of both anions in the eluate was exactly the same as in the feed, so reaching the breakthrough point.

The selective removal of chloride with respect to AA depends on two factors, the intrinsic affinity of the resin for them and the pH. As the affinity of the resin for chloride is higher than for acetate, chloride can displace acetate anions bound to the resin. So, as the liquid front moves through the column, when it finds free binding sites, both chloride and acetate can be bound. However, when the liquid behind the front finds that the binding sites are occupied, acetate can not bind and continues its way to the next free sites, but chloride can displace the acetate previously bound to the resin. As a result, the liquid front would be depleted in chloride and enriched in acetate, which explains the elution pattern showing an overshooting of acetate after the breakthrough of chloride.^24^

In addition, pH also plays a relevant role in this process. The pH of the first fraction of eluate increased abruptly from the initial pH of the DM-DPM medium (2.95) to 4.7 and then decreased slowly in the next fractions, as the feed passed through the column, to finally reach the pH value of the feed. As repeatedly explained previously, the charged acetate fraction depends on the pH of the medium, so that the higher the pH the more acetate will be present. This means that at the initial BV, when the pH is higher, more acetate molecules are available for binding. Later, as the pH decreased, most of AA molecules would be undissociated (uncharged) and unable to bind to the resin. Therefore, this pH effect would enhance the displacement of acetate by chloride.

If all the fractions eluted to the breakthrough point of chloride are pooled, the resulting solution would contain 92% of the AA fed to the column and one third of the chloride, so obtaining a solution enriched in AA with respect to the DM-DPM medium, but the purification parameters (Table 3) would not improve the results obtained in batch mode with the same resin.

By discarding some of the fractions eluted at both extremes of the breakthrough curve, the purity and enrichment factor of the process would improve, but it would be detrimental for the recovery yield, which would decrease to an unacceptable degree.

The reason behind the worse performance obtained in column compared to batch might be related to the influence of the flow rate of the liquid through the column on the binding of the anions to the resin. The “contact time” between the anions and the binding sites in the column would decrease at higher flow rates, surpassing its kinetic capabilities, so being more difficult to reach equilibrium and likely negatively affecting anion separation.^25^ So, a low flow rate would be preferred. In the column experiment the residence time was 1 min, so that the “contact time” was quite short. A lower flow rate could also be applied, but the time required to pass the liquid would be extremely high. For example, in the column system used in this study, it would be necessary 500 min (more than 8 h) to pass 500 mL of liquid. If the flow rate is reduced by half, the time would be increased the double, to 1000 min (more than 16 h), which would be operationally unpractical. Under batch mode, conversely, the “contact time” is higher, high enough to allow equilibrium to be reached, and independent of the liquid volume to be treated.

Another factor that could be involved in the lower performance achieved under column mode could be the nature of the mixed bed resin. This kind of IEX resin contains a mixture of strong acid cation and base anion exchange resins and, according to the manufacturer, the densities of both resins are quite different, being lower that of the latter. This means that during the resin bed formation in the column some degree of separation of the resins could have occurred, resulting in an uneven distribution of both types of resin, with the base anion exchange resin enriched towards the top of the column and *vice versa*. And this uneven distribution of the resins could locally affect both the binding of ions and pH, which are closely linked, thus affecting the beneficial pH-buffering effect of the mixed bed resin and resulting in a lower separation performance than expected if the resins had been distributed homogeneously throughout the column. Under batch mode, however, this phenomenon would not occur and the separation achieved with the mixed bed resin would be better.

Although the purification of AA under the conditions of scenario 1 was considerably improved, particularly under batch mode, the acid still remained in solution at a very diluted concentration, even lower than in the original DPM medium. Accordingly, it would be necessary to apply additional treatments to fully recover and concentrate AA, which would reduce again the recovery yield and make the process unfeasible. So, a different approach was required to further improve purification, and this is where scenario 2 appears.

#### 3.2.4. Recovery and purification of AA with Amberlite MB20 in batch – step-elution with H_2_SO_4_

The previous experiments showed a better performance for the Amberlite MB20 resin in batch mode than in column mode. So, an AA recovery and purification strategy based on the scenario 2 described in Section 3.2.2 under batch mode was assessed. The idea was to first remove AA and chloride totally from DM-DPM medium with Amberlite MB20 in batch (5 g L^−1^) and then selectively elute AA using a small volume of a diluted solution of sulfuric acid. Elution was carried out in batch, by successively applying small volumes of the eluent, so that it would be a step-elution.

There were several reasons to apply such kind of step-elution with sulfuric acid. First, considering the affinity order of the resin for the anions (sulfate > chloride > acetate), it was expected that sulfate eluent would first displace acetate from the resin and later chloride. Second, the acidic pH of the sulfuric acid solution would shift the AA/acetate equilibrium to the formation of undissociated AA, which would enhance its release from the resin binding sites. Third, the step-elution under batch mode would allow to reach the binding equilibrium of all the anionic species involved by simply extending the “contact time” sufficiently (a “contact time” of 30 min was found to be enough to reach equilibrium). Fourth, the step-elution would allow precise control of the extent of the acetate displacement and elution, allowing the elution to be finished when chloride or sulfate began to appear in the eluate. And fifth, the use of small volumes of eluent would allow to obtain a more concentrated solution of AA in the eluate compared with that in the feeding.

**Fig. 6:**
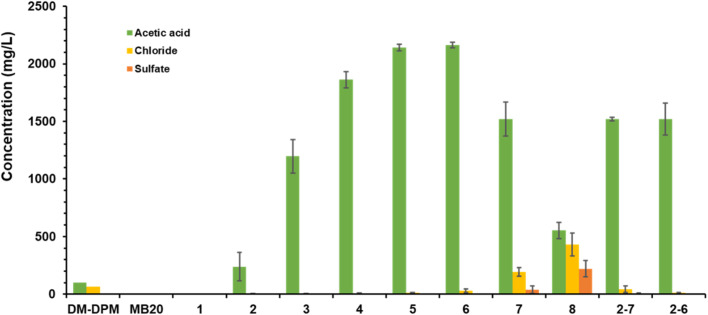
Recovery and purification of AA with Amberlite MB20 in batch involving a step-elution with 20 mM H_2_SO_4_. DM-DPM, the original medium; MB20, DM-DPM medium after treatment with the Amberlite MB20 resin; 1 to 8, elution steps; 2–7, pooled fractions from elution steps 2 to 7; 2–6, pooled fractions from elution steps 2–6.

A 500 mL solution of DM-DPM medium was treated with Amberlite MB20 at a rate of 5 g L^−1^ at room temperature for 2 h with gentle stirring to remove completely AA and chloride. Then, the anion-loaded resin was separated from the anion-depleted liquid by filtration. The anion-loaded resin was finally step-eluted with 20 mM H_2_SO_4_ applied in eight 5 mL steps. Each elution step involved the addition of 5 mL of the eluent to the resin, stirring for 30 min, and separation of resin and liquid by filtration. The results of this process are shown in Fig. 6.

Treatment of the DM-DPM medium with the resin resulted in the complete removal of both AA and chloride, leaving a liquid that was essentially pure water, that could be further reused supporting the sustainability of the process.

The anion-loaded resin was then step-eluted. In the first elution fraction (1) no anions were detected, which suggest that sulfate anions had bound to free binding-sites still present in the resin. Thereafter, in the next four elution steps (2–5), the AA concentration in the elution fractions steadily increased, reaching a maximum value as high as nearly 2200 mg L^−1^ in the step 5, that is, more than 20 times more concentrated than in DM-DPM medium. Chloride, in turn, was hardly detected in these fractions, with concentrations lower than 15 mg L^−1^ in all of them, and sulfate was totally undetectable. From step 6, AA concentration began to decrease and, at the same time, concentration of chloride, first, and sulfate, later, increased.

If fractions 2 to 7 (2–7) are pooled the resulting solution would contain 1520 mg L^−1^ of AA and only 42 mg L^−1^ of chloride, with a recovery yield for AA of 90.3%. This means that AA would have been concentrated by around 15 times, while chloride levels would be 37% lower than in the original DM-DPM medium, so having considerably improved its purity. Instead, if those that are pooled are fractions 2 to 6 (2–6), the AA concentration would be the same, 1520 mg L^−1^, and that of chloride lower, 12 mg L^−1^, that is, a greater purity would be obtained, but with a lower recovery yield of 75%. The increase in the concentration of AA in the pooled elution fractions compared to that in the original DM-DPM medium results from the strong decrease in the volume of the solutions, from 500 mL to 30 or 25 mL for pooled fractions 2–7 or 2–6, respectively.

The purification parameters of this process are shown in Table 4. The AA purity of the pooled fractions 2–7 and 2–6 would be 96.9 and 99.2%, respectively, so clearly improving the values obtained in the previous processes, involving treatments with the same resin (scenario 1) under batch or column modes. Moreover, as a result of the purity improvement, the enrichment factor shot up to values as high as 1256 and 5086, respectively. The aggregate recovery yield was the only parameter with lower data: slightly lower, but not significantly different, for the pooled fractions 2–7 (82.2 *vs.* 83.7%), and 18% lower for pooled fractions 2–6.

**Table 4:** Performance of the purification process of AA involving treatment of DM-DPM medium with the MB20 resin in batch (scenario 2) and a step-elution with H_2_SO_4_

Parameter	DPM	DM-DPM	DM-DPM MB20 (2–7)^c^	DM-DPM MB20 (2–6)^d^
Yield (%) step^a^	100	91.0	90.3	75.3
Yield (%) aggr.^b^	100	91.0	82.2	68.5
Purity (%)	2.4	59.8	96.9	99.2
Purity increase	1.0	24.6	40.4	41.3
Enrichment factor	1.0	58	1256	5086

a Yield in a step with respect to the previous one.

b Aggregate yield in a step with respect to the starting DPM medium.

c MB20 (2–7), pooled fractions from elution steps 2 to 7.

d MB20 (2–6), pooled fractions from elution steps 2 to 6.

**Fig. 7:**
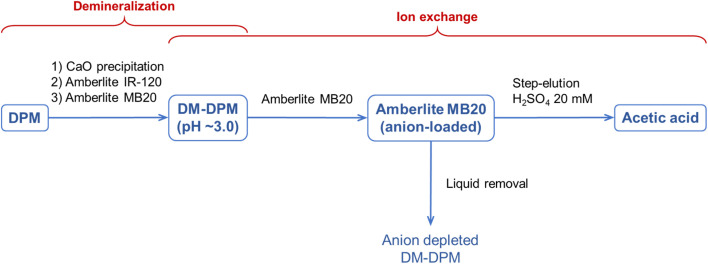
Scheme of the proposed whole recovery and purification process of AA from DPM medium, involving successive steps of demineralization, treatment with the Amberlite MB20 resin in batch and step-elution with H_2_SO_4_.

Furthermore, apart from the better results regarding purity and enrichment, the purification process described in this section had an additional and very relevant benefit: the final AA solution was concentrated by 15–16 times compared to the original DPM medium, while in the other two processes its concentration was around 10% lower. Therefore, further concentration of AA to industry-demanding levels using conventional technologies, preferably non-energy intensive technologies such as liquid–liquid reactive extraction^26^ or IEX resins again, would be easier by applying this process. A scheme of the whole recovery and purification process proposed in this work is presented in Fig. 7.

## 4. Conclusions

In this paper, a case study dealing with the technical feasibility of a downstream process for the recovery and purification of AA from extremely diluted solutions (100 mg L^−1^ or 0.01% w/w) containing contaminating inorganic salts is presented. The process is based on two successive steps using of IEX resins, that is, a non-energy intensive separation technology. The first step, demineralization, involved a combined treatment of calcium precipitation, acidification with the Amberlite IR-120 resin and treatment with the mixed bed Amberlite MB20 resin, which allowed the total removal of phosphate and sulfate (and likely bicarbonate) and 90% removal of chloride, while still remaining 91% of AA in solution. The demineralized medium resulting from this first step was, in the second step, treated again with the mixed bed Amberlite MB20 resin in batch to remove all AA and chloride remaining in solution and, finally, the anion-loaded resin was step-eluted with a low volume of diluted H_2_SO_4_ to selectively elute AA. The recovery yield and purity of AA in the final solution obtained showed an inverse relationship depending on the number of eluted fractions pooled. The greater the number of fractions pooled (2–7 *vs.* 2–6), the greater the recovery yield (82.2 *vs.* 68.5%) but the lower the purity (96.9 *vs.* 99.2%). In any case, the values of both parameters appear to be good, especially considering the final solution of AA obtained, which was 15-fold more concentrated than the original medium (>1500 *vs.* 100 mg L^−1^).

Two issues should be highlighted to support the novelty of this work. On the one hand, the vast majority of downstream processes dealing with the recovery of AA, or carboxylic acids in general, from fermentation media are applied to solutions with concentrations of AA, at least, one to two orders of magnitude higher than the concentration available in this work. On the other hand, a mixed bed ion exchange resin is used in this work to both demineralize the AA solution and purify it, instead of the commonly used single strong or weak base anion exchange resins. As far as we know there are no reports in the literature addressing the recovery and purification of AA (or other short-medium chain length fatty acids) either from extremely diluted solutions nor using mixed bed ion exchange resins.

It is worth mentioning that although the experimentation has been done with synthetic solutions the results can be fully extrapolated to real samples such as broths resulting from CO_2_ fermentation processes to AA, characterized by the very low content of the acid. The microbial biomass present in the broth would be easily removed by microfiltration or centrifugation, and the macromolecular compounds contained in the clarified broth by ultrafiltration. The resulting broth would mainly contain AA and the inorganic salts, so it would be very similar to the DPM synthetic medium used in this work. Other compounds potentially present in the broth, such as trace elements and vitamins, would be at so low concentrations that would hardly interfere with the purification process.

## Data availability

The data associated with this article have been included in the manuscript.

## Author contributions

Tomás Roncal: conceptualization, methodology, investigation, formal analysis, data curation, writing – original draft, writing – review & editing, visualization, supervision, project administration. Ainhoa Aguirre: investigation, resources, data curation. Yolanda Belaustegui: writing – review & editing, funding acquisition, project administration. Elisabet Andrés: resources, project administration.

## Conflicts of interest

There are no conflicts of interest to declare.
